# A Multi‐Level Intervention to Address Childhood Obesity in Rural Hispanic Communities

**DOI:** 10.1002/osp4.70116

**Published:** 2026-01-23

**Authors:** Linda K. Ko, Eileen Rillamas‐Sun, Mario Kratz, Eligio Jimenez, Sou Hyun Jang, Jason A. Mendoza, Sonia Bishop, Lan Xiao

**Affiliations:** ^1^ Department of Family Medicine Keck School of Medicine University of Southern California Alhambra California USA; ^2^ Department of Health Systems and Population Health University of Washington and Public Health Sciences Division Fred Hutchinson Cancer Center Seattle Washington USA; ^3^ Division of Public Health Sciences Fred Hutchinson Cancer Center Seattle Washington USA; ^4^ Community Safety Network Toppenish Washington USA; ^5^ Department of Sociology Korea University Seoul Korea; ^6^ Division of Public Health Sciences Fred Hutchinson Cancer Center and Department of Pediatrics University of Washington Seattle Washington USA; ^7^ Department of Health Systems and Population Health University of Washington Seattle Washington USA; ^8^ Department of Epidemiology and Population Health Stanford University Stanford California USA

**Keywords:** childhood obesity, Hispanic, multi‐level intervention, rural communities

## Abstract

**Objectives:**

Pediatric obesity disproportionately affects children of lower socioeconomic status, racial and ethnic minorities, and rural communities, and is influenced by social and physical environments. Community‐engaged interventions can address pediatric obesity and have been implemented in rural settings for other conditions, but few have specifically targeted rural childhood obesity. Together We STRIDE study is a community‐based trial designed to test the effectiveness of a multi‐level obesity prevention intervention in Hispanic children living in rural communities.

**Methods:**

The trial enrolled 653 children (8–12 years old). The 13‐month (March 2017–April 2018) multi‐level intervention included comic books, nutrition and physical activity (PA) classes, media literacy education and PA breaks, and an open‐street community program (Ciclovía). The primary outcome was between‐group differences in BMI *z*‐score, measured at baseline, 6 months, and 18 months.

**Results:**

There were no significant between‐group differences in BMI *z*‐scores and BMI‐for‐age percentile relative to 95th percentile at 6 months or 18 months follow up. The mean difference in BMI *z*‐score between intervention and comparison communities was −0.02 (95% CI −0.05, 0.02; *p* = 0.31) at 6 months and 0.03 (95% CI −0.03, 0.09; *p* = 0.32) at 18 months, respectively. BMI *z*‐scores decreased progressively with increased exposure to intervention components (unadjusted *p*‐trend = 0.008 and adjusted *p*‐trend = 0.009).

**Conclusions:**

Although this multi‐level community‐based intervention did not show an overall intervention effect on BMI *z*‐scores, greater engagement with the intervention components was associated with higher reductions in BMI *z*‐scores. The findings underscore both the promise and the challenges of community‐based obesity prevention interventions in rural communities.

**Trial Registration:**

NCT02982759 (Together We STRIDE) retrospectively registered during study recruitment

## Graphical abstract

Together We STRIDE study is a community‐based trial designed to test the effectiveness of a multi‐level obesity prevention intervention in Hispanic children living in rural communities. The trial enrolled 653 children. Although this multi‐level, community‐based intervention did not show an overall intervention effect on BMI *z*‐scores, greater engagement with the intervention components was associated with higher reductions in BMI *z*‐scores.

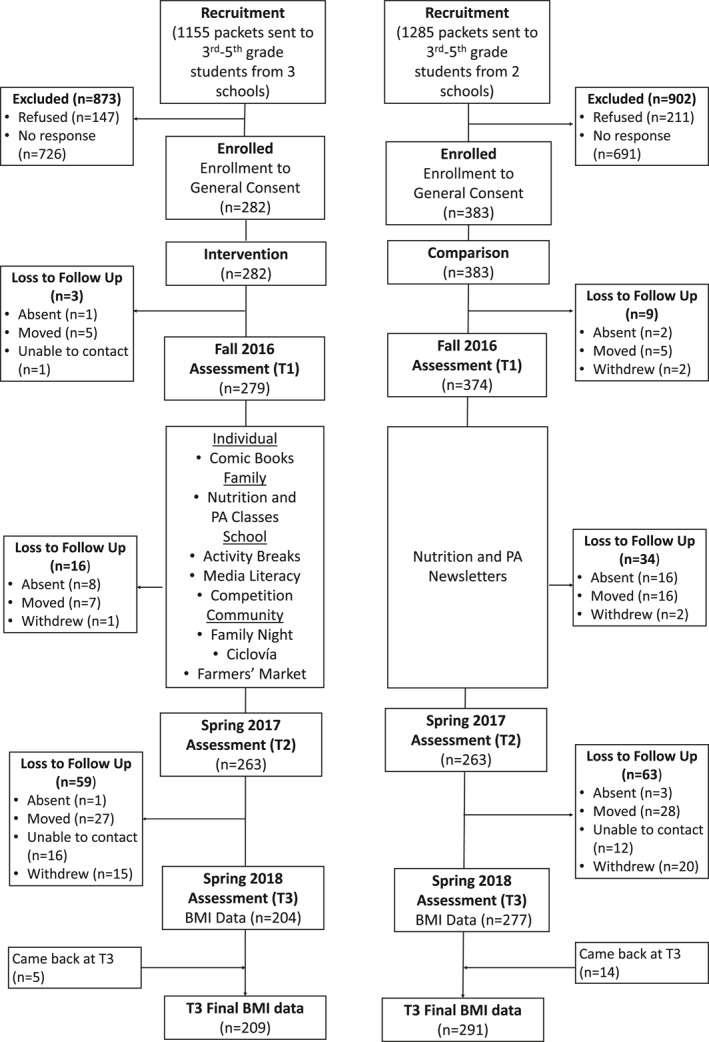

**Abbreviations**
BMI: Body Mass Index
CAB: Community Advisory Board
CBPR: Community‐based Participatory Research
CDC: Centers for Disease Control and Prevention
CHW: Community Health Worker
CI: Confidence Interval
DSQ: Dietary Screener Questionnaire
PA: Physical Activity
RUCA: Rural‐Urban Commuting Area
SD: Standard Deviations
STRIDE: Strategizing Together Rural Interventions for Diet and Exercise

## 1 Introduction

Pediatric obesity is a pervasive threat to health that disproportionately affects children of lower socioeconomic status [1, 2], racial and ethnic minorities [3, 4], and rural communities [5]. Such disparities reflect the interesting influences on children's healthy eating and physical activity (PA) behaviors. For example, social and physical environments including neighborhood poverty [6], limited access to recreational facilities [7, 8], low sidewalk availability [9, 10], and healthy restaurants and grocery stores are major determinants of pediatric obesity [11, 12, 13], as are the community, schools, peers, and family environment [11, 12, 14]. Rural communities in the U.S. and abroad face unique environmental challenges to obesity prevention due to geographic isolation, lack of transportation infrastructure, and limited access to preventive healthcare [14, 15, 16]. These challenges are compounded for Hispanic families living in rural areas, who may also experience language differences, limited access to culturally responsive services and foods, low wage employment, and immigration‐related stressors, all of which impact diet and PA [16].

Existing research on obesity prevention in rural communities has primarily focused on changing the family home environment with mixed success [17, 18, 19]. For instance, a 10‐month intervention among 114 parent‐child dyads (ages 7–10) in rural Minnesota that delivered education and goal setting sessions to improve the home food environment [17] found no significant effect on BMI or obesity, despite trends in the hypothesized directions. Moreover, a recent review of obesity prevention intervention studies found that family‐level interventions in Hispanic communities improved obesity‐related health behaviors and outcomes, but only nine studies met the review criteria for cultural relevance [20].

Conversely, school‐based community interventions have demonstrated positive impacts on children's BMI, including specific studies focused on Hispanic youth in rural areas [21]. For example, a multi‐component school‐based intervention developed through researcher‐school district partnerships in California's rural central valley significantly improved BMI indicators among 782 children (ages 3–8) of Mexican heritage. An overview of Cochrane reviews on pediatric obesity concluded that school‐age children may be more influenced by the obesogenic environments than younger children [22], highlighting the need for community‐based interventions among school‐age children. These findings suggest that supporting children's healthy food and PA choices requires multi‐level interventions that extend beyond children to engage parents, schools, and the broader community. Given the limited infrastructure around nutrition and PA in rural Hispanic communities, a community‐engaged approach could galvanize community resources and create community‐wide healthy norms to prevent obesity [9, 10, 11, 12, 13].

A community‐based participatory research (CBPR) builds on the complementary strengths and insights of community and academic partners to collaboratively identify systematic inquiries, participate in the research process, and take action to address health problems [23, 24, 25]. CBPR approaches, particularly those that integrate community health workers (CHWs) and address children's social and physical environment, show promise for obesity prevention and control [6, 9]. For example, a systematic review found that various CHW roles in community‐based obesity interventions made small but significant improvements in the BMI *z*‐score and BMI percentile of children from underserved populations [6, 9]. A 3‐year CBPR multi‐level intervention study of 1858 kindergarten–fifth grade students conducted in Massachusetts significantly decreased children's body mass index (BMI) *z*‐scores and obesity prevalence [26]. Similarly, a 2‐year CBPR intervention that addressed multi‐level environmental factors among 1028 students in grades 1–3 decreased BMI *z*‐scores and rates of overweight and obesity [27]. Together, these studies demonstrate the potential of CBPR for addressing pediatric obesity [26, 27].

Together We STRIDE (Strategizing Together Rural Interventions for Diet and Exercise; herein STRIDE) was implemented to build upon these findings by testing the effectiveness of a community‐wide, multi‐level intervention on BMI among rural Hispanic children living in Washington State.

## 2 Methods

### 2.1 Study Setting

This CBPR study builds on over two decades of partnership between researchers and local community advisory boards (CABs) in Eastern Washington State's Lower Yakima Valley, where, in 2022, Hispanics comprised over half of the population [28]. A Yakima Valley community‐wide needs assessment identified pediatric obesity as a key health priority [29, 30]. CAB members, who represent diverse community sectors including social services, community health centers, faith‐based organizations, and schools, assisted in the identification of specific communities within the Valley to engage in the STRIDE intervention.

### 2.2 Design Overview

This quasi‐experimental study took place from January 2016 to December 2019. It included two rural communities (one intervention and one comparison). Both communities are classified as a large rural community, based on the Rural‐Urban Commuting Area code of 4.2, and have a population of approximately 10,000 people, 74% of whom are Hispanic, and 24% of whom are living below the federal poverty level. Both communities have a comparable number of elementary schools, school size, and student characteristics (i.e., standardized test scores and percentage of students eligible to receive free or reduced‐price lunch) [31].

As described in the protocol and previously published data [10, 13, 29], CAB members who represent both communities, collaboratively reviewed the needs assessment data and assigned the community they perceived to have fewer resources (e.g., access to fresh food, safe outdoor recreation space, and employment opportunities) as the intervention community. Selection occurred through anonymous voting by CAB members; research team members did not vote.

### 2.3 Recruitment

**FIGURE 1:**
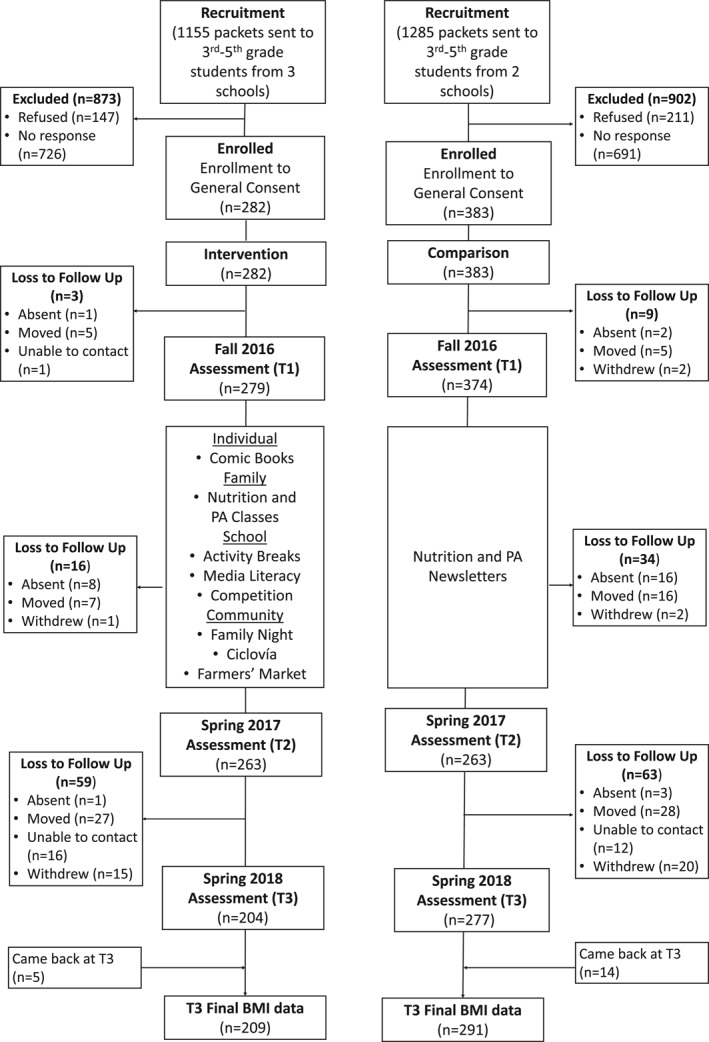
Participant flow diagram.

The participant flow diagram (Figure 1) shows participant recruitment, screening, measurement, and retention data. Children were eligible if they (1) identified as Hispanic, (2) were in grades 3–5 (ages 8–12 years), and (3) attended an elementary school in the intervention or comparison communities. To ensure safety and intervention feasibility, children were excluded if they had a medical condition that would prevent safe participation in the intervention's PA or required specialized medical diets, as reported by their parent.

Children were recruited from five elementary schools (three schools from the intervention community and two schools from the comparison community). Packets containing information about the study, a parent consent form, a student assent form, and a screener form were distributed to children in grades 3–5 through their teachers; documents were in both English and Spanish. Parents interested in enrolling their child(ren) signed informed consent and completed a screener survey to document their child's grade level, race/ethnicity, health insurance and household income. Children were enrolled upon confirmation of eligibility and receipt of parental's signed consent and child's written assent. Of the 1023 students screened as eligible, 653 (63.8%) enrolled in the study; 279 (65%) in the intervention arm and 374 (63%) in the comparison arm.

### 2.4 Intervention

STRIDE included intervention activities at multiple levels of the children's environment (Table 1) [29]. At the *individual level,* children received four comic books with targeted messages about healthy eating and PA. The comic books follow a fifth‐grade boy who discovers superpowers through eating fruits and vegetables and being physically active. Two comic books were distributed annually over the 2‐year intervention.

**TABLE 1:** Intervention components of the Together We STRIDE.

Multi‐level components	Intensity	Description
Children‐facing comic books	4 episodes delivered 2 per intervention year	Hector and the Mystery of His Power Book 1: Hector is introduced to Sam & Hannah, two students he has never seen at school before. Book 2: Hector eats a super fruit and feels stronger and agile. Sam tries to shrug it off. Book 3: Hector finds fruits and vegetables missing from stores and parks are disappearing and learns Sam and Hannah are taking them all to their own planet. Hector eats his super fruit (peach) and flies after Sam and Hannah's spaceship. Book 4: Hector learns what happened to Sam and Hannah's planet when another planet introduced them to technology making them more sedentary and sets out to their planet after showing all his friends how to find their own superpowers from eating fruits and vegetables.
Family‐facing nutrition and PA classes delivered by community health workers	8 modules delivered in year 2 of intervention	Module 1: Our Values: Our Health Module 2: Physical Activity is Key to Living Well Module 3: Catch a Rainbow Every Day Module 4: What is on your plate? Part 1 Module 5: What is on your plate? Part II Module 6: To Be or Not to Be Organic Module 7: Kitchen Gardens Module 8: Eating Healthy on a Budget
Media literacy delivered by teachers	3 lessons per year	Brief 10–15‐min lessons focused on how to respond critically and thoughtfully to food advertising and screen time. Lesson 1: Food Packaging Lesson 2: Food Portions Lesson 3: Marketing Appeal Lesson 4: Technology and You Lesson 5: Screen Time and Physical Activity Lesson 6: Kid Food vs Adult Food Lesson 7: Eat a Rainbow Lesson 8: Food Product Placement in Movies Lesson 9: Ask Your Parents for Help Lesson 10: What Can Your Community Do
Physical activity breaks delivered by teachers	4 sessions per month	Teachers had access to GoNoodle Plus to incorporate physical activity sessions into math, writing, reading, geography, science, and technology. GoNoodle activities included: Activity 1: Mega Math Marathon Activity 2: Bodyspell Activity 3: Word Jam Activity 4: Freeze It Activity 5: Montana James and the Palace of Peril Activity 6: Field Trip Activity 7: Think on Your Feet Activity 8: My Questions Activity 9: Ultimate Champ Training Activity 10: Flow and Think About It
Annual open‐streets programming of Ciclovía	Annually	The community advisory board and the intervention city collaborated with the research team to organize the Ciclovía, closing streets around an adjacent park for walking, biking, jogging, and playing various sports and physical activities.

At the *family level,* children and their families attended multi‐generational nutrition and group PA classes led by community health workers (CHWs) in a community setting and were offered in the second year of the intervention. Eight classes were delivered weekly over 8 weeks. The topics included (1) a discussion about values and health, (2) types of PA and ways to incorporate PA every day, and (3) a discussion about MyPlate. Each class session included (1) an icebreaker activity, (2) nutrition and PA education, (3) a cooking demonstration, (4) group PA, and (5) weekly goal setting around healthy food choices and/or PA.

At the *school level,* teachers in grades 3–5 integrated media literacy education and PA breaks into their classroom curriculum. Media literacy education was designed to help students respond critically to advertising messages and images of food products [32, 33, 34]. Teachers were asked to cover three lessons (out of 10) per school year. Each grade received the same lesson content, with different grade‐appropriate discussion questions. For example, in Lesson 1, “Food Packaging,” children viewed different packages of food products (e.g., Cheetos, Pop tarts, etc.) and participated in a teacher‐moderated discussion. Discussion questions for third graders included: “What words and images do you see?” “What do the words and images try to tell you?” Discussion questions for students in grades 4–5 included: “How do these products pretend to be healthy?” “What are some other snacks that would be a healthier option?” Teachers were also asked to integrate one PA break (3–10 min sessions) per week into their curriculum [35].

*Community‐level* activities included a Family Night and Ciclovía. The Family Night was a onetime intervention launch event in March 2017 that brought families together to share a healthy meal, learn about nutrition and PA, engage in a discussion about obesity prevention, and participate in group PA. Ciclovía held annually in Spring 2017 and 2018 enabled community members to “claim the streets” for PA, such as walking and cycling. The CAB selected the location for the Ciclovía, obtained a permit from the city to close the streets, and recruited volunteers to lead activity hubs for adults (e.g., aerobics, yoga, and Zumba) and children (e.g., hula hoops, foursquare, and a bike‐riding course) [29].

### 2.5 Comparison Group

Children in the comparison community received two generic health‐based newsletters. The newsletters contained information about the benefits of healthy eating and PA, goal setting, and a list of resources on healthy eating that are publicly available on the internet.

### 2.6 Data Collection

Demographics, height and weight were collected for all children (*n* = 665). Height and weight assessments at baseline, six and 18 month were conducted at participants' schools by grade level. Six stations were set up in a classroom to measure six students at the same time. To promote privacy at each station, a large color poster blocked the scale from other students. Students who were absent on measurement days were contacted by phone to be measured at a place of their preference. Eighteen‐month data collection for fifth graders at enrollment was conducted outside of school as they had moved to middle school. Participants received a small gift (worth $3) for their participation.

Children were weighed in light clothing (without shoes) to the nearest 0.1 kg using a portable Tanita WB‐110A digital body weight scale (Tanita Corporation America, Arlington Heights, IL, 2001). Height was measured to the nearest 0.2 cm using a Charder HM200P Portstad Portable Stadiometer (Charder Medical, Taiwan, ROC, 2007). BMI *z*‐scores and BMI‐for‐age percentiles relative to the 95% percentile, an established measure of severe obesity, were also calculated using a Center for Disease Control (CDC) coded SAS (SAS Institute Inc., Cary, NC, USA) program (version released 11/2016) [36]. The SAS programming code calculates *z*‐scores, percentiles, and BMI as a percentile of the 50th and 95th percentiles based on the 2000 CDC growth charts [36].

### 2.7 Statistical Analysis

Descriptive statistics for children's demographic and socioeconomic characteristics were calculated. Distributions were tabulated and Chi‐square tests were used to compare between‐intervention arm differences. Means and standard deviations (SD) of BMI *z*‐scores and BMI‐for‐age percentiles relative to the 95% percentile at baseline, 6 months, and 18 months were estimated for all children.

#### 2.7.1 Intervention Effect

The community effect and community × time interactions were evaluated using repeated‐measures and mixed‐effects linear models from all participants with BMI data at follow‐up. The fixed effects of each model included baseline values of BMI measures, sex, age, ethnicity, community (intervention or comparison), time point (six and 18 months), and community × time interactions. The community × time interactions tested the between‐community differences at the 6 and 18‐month follow‐up. Given the similar communities between intervention and comparison, the baseline covariates should most likely be comparable and thus not bias the results. As they are associated with the outcomes, adjusting for baseline covariates in the analysis may improve statistical efficiency [37]. The random effects accounted for repeated measures with an unstructured covariance matrix and the clustering of students within families and within classes. Missing data were handled directly through maximum likelihood estimation via mixed modeling.

Although fifth graders were recruited and enrolled, they were not exposed to the full 2‐year intervention. After graduating from their elementary school Year 1, they continued receiving comic books and attended the Family Classes, and Ciclovia, but did not receive the school level intervention components of media literacy and PA breaks. Therefore, a sensitivity analysis excluding fifth grade students was conducted. An additional sensitivity analysis excluding children who had outliers for the outcomes was also conducted. The moderation analysis used the same models mentioned above but included an interaction of each potential effect modifier (i.e., baseline obesity status, sex, or grade) with community; if the interaction term was significant, the null hypothesis of no moderation was rejected.

#### 2.7.2 Exposure

As a post hoc analysis, the study team examined whether more exposure to the four intervention components (i.e., comic books, family classes, activity breaks, and media literacy classes) was associated with changes in BMI outcomes.

Each comic book was placed in a labeled plastic bag (student name, teacher's name, and grade) and given to intervention schoolteachers to distribute to students. The comic book score component was calculated as the proportion of the two comic books received each year of the intervention, based on self‐report: 0 (none), 0.5 (one of two), and 1 (both received). The family classes’ component score was calculated as the proportion of the eight classes attended.

Teachers tracked their delivery of activity breaks and media literacy classes using poster tallies, which were collected and tabulated at the end of the school year. The activity breaks component score was calculated as the proportion of the 11 possible total breaks administered in Year 1 and the proportion of 30 possible total breaks administered in Year 2. The media literacy component score was calculated as the proportion of three classes delivered, as teachers were instructed to deliver at least three media literacy lessons per school year.

These individual component scores were then used to calculate a total intervention exposure score by dividing by three for Year 1 intervention components (comic books, activity breaks, and media literacy classes) and by 4 for Year 2 intervention components (addition of family classes). The analysis assigned each child an exposure composite score ranging from 0 (no exposure to any intervention component) to 1 (full exposure to all four components). The total intervention composite exposure score was categorized into 5 groups based on level of exposure: 0, > 0 to 0.25, > 0.25 to 0.5, > 0.5 to 0.75, and > 0.75 to 1. The analysis applied exposure score categories to determine the mean change in the measures at the 6 and 18‐month follow‐up for the exposure scores of the intervention. To test whether more exposure to the intervention components was associated with greater changes in BMI measures at follow‐up, linear regression models that included the exposure score as an ordinal variable (range 0–4) were used, allowing a test for trend. These models also included adjustment for BMI measures at baseline.

As estimated, 600 participants (300 per intervention arm) would be needed to provide 80% power to detect a difference of 0.23 change in BMI *z*‐scores in the intervention community compared to the comparison community, assuming a 2‐sided α level of 5% and 67% retention. All analyses were conducted using SAS version 9.4 (SAS Institute Inc., Cary, NC, USA). Statistical significance was defined as a 2‐sided test of < 0.05.

## 3 Results

### 3.1 Participant Demographics

Table 2 shows the demographics of the children by community (*N* = 653; 279 intervention and 374 community). Enrollment of children in grades 3 (36%), 4 (33%), and 5 (31%) was nearly evenly distributed. About half of the children (49%) were male; majority (87%) were Hispanic; 83% had public health insurance; 52% were from families reporting a yearly income of $35,000 or less. Demographic characteristics did not differ statistically significantly between intervention and comparison communities.

**TABLE 2:** Baseline characteristics of participants in intervention and comparison community in the Together We STRIDE trial: Yakima County, WA, 2016–2018.

Characteristic	Total	Intervention community	Comparison community	*p*‐value
*N* (%)	653	279 (42.7)	374 (57.3)	
Language for screener, *n* (%)				0.14
English	355 (54.4)	161 (57.7)	194 (51.9)	
Spanish	298 (45.6)	118 (42.3)	180 (48.1)	
Grade level, *n* (%)				0.43
3	237 (36.3)	102 (36.6)	135 (36.1)	
4	215 (32.9)	85 (30.5)	130 (34.8)	
5	201 (30.8)	92 (33.0)	109 (29.1)	
Sex, *n* (%)				0.48
Male	317 (48.6)	131 (47.0)	186 (49.7)	
Female	336 (51.5)	148 (53.1)	188 (50.3)	
Ethnicity, *n* (%)				0.23
Hispanic	569 (87.1)	238 (85.3)	331 (88.5)	
Non‐Hispanic	84 (12.9)	41 (14.7)	43 (11.5)	
Insurance, *n* (%)				0.2
None	8 (1.3)	3 (1.1)	5 (1.4)	
Private (e.g., Premera, Blue Cross)	103 (16.3)	36 (13.3)	67 (18.5)	
Public (e.g., Apple Health, IHS)	523 (82.5)	232 (85.6)	291 (80.2)	
Household Income, *n* (%)				0.25
< $15,000	102 (17.1)	42 (16.3)	60 (17.8)	
$15,000 to < $35,000	207 (34.7)	92 (35.7)	115 (34.0)	
$35,000 to < $50,000	97 (16.3)	44 (17.1)	53 (15.7)	
$50,000 or more	89 (14.9)	30 (11.6)	59 (17.5)	
Don't know^a^	101 (17.0)	50 (19.4)	51 (15.1)	

*Note:* missing data non‐Hispanics (*n* = 1); Insurance (*n* = 19), and income (*n* = 57).

^a^ Included for descriptive purposes only; was not included in the analysis of difference.

### 3.2 BMI *z*‐Score

Table 3 shows the primary outcomes at the three time points (baseline, 6 months, and 18 months) among intervention and comparison communities. The mean difference in BMI *z*‐score was 0.01 (95% CI −0.04, 0.05; *p* = 0.78) among children in the intervention community relative to the comparison community. The mean difference in BMI *z*‐score at 6‐ and 18 month between the intervention and comparison community was −0.02 (95% CI −0.05, 0.02; *p* = 0.31) and 0.03 (95% CI −0.03, 0.09; *p* = 0.32), respectively.

**TABLE 3:** Differences in body mass index measures at 6‐ and 18 month comparing intervention and comparison communities: The Together We STRIDE trial, Yakima County, WA, 2016–2018.

	Total	Raw estimates		Treatment effect^a^	
		Intervention community	Comparison community	Adjusted between‐group difference (95% CI)	*p*‐ value
*N* (%)					
Baseline (T1)	653	279 (42.7)	374 (57.3)		
6 months (T2)	603	263 (43.6)	340 (56.4)		
18 months (T3)	500	209 (42.8)	291 (58.2)		
BMI, kg/m^2^, mean (SD)					
Intervention effect				0.07 (−0.13, 0.27)	0.51
Baseline (T1)	21.0 (5.0)	21.3 (5.5)	20.8 (4.6)		
6 months (T2)	21.4 (5.0)	21.4 (5.0)	21.4 (5.0)	−0.05 (−0.21, 0.12)	0.59
18 months (T3)	22.6 (5.6)	22.5 (5.6)	22.7 (5.7)	0.18 (−0.11, 0.48)	0.23
BMI‐for‐age *z*‐score, mean (SD)					
Intervention effect				0.01 (−0.04, 0.05)	0.78
Baseline (T1)	1.03 (1.05)	1.05 (1.06)	1.01 (1.05)		
6 months (T2)	1.01 (1.11)	1.00 (1.05)	1.02 (1.15)	−0.02 (−0.05, 0.02)	0.31
18 months (T3)	1.07 (1.03)	1.04 (1.04)	1.10 (1.02)	0.03 (−0.03, 0.09)	0.32
BMI‐for‐age percentile relative to 95th percentile, mean (SD)					
Intervention effect				0.46 (−0.39, 1.31)	0.29
Baseline (T1)	94.5 (22.0)	95.5 (23.7)	93.9 (20.7)		
6 months (T2)	93.9 (21.7)	93.5 (21.5)	94.3 (21.7)	−0.08 (−0.80, 0.63)	0.82
18 months (T3)	94.8 (23.7)	94.2 (23.4)	95.3 (23.9)	1.00 (−0.23, 2.23)	0.11

Abbreviations: BMI, body mass index; SD, standard deviation; T, time.

^a^ Linear mixed models accounting for the random effects of the repeated measures and clustering of students within families and students within classes. Models adjusted for baseline outcome value, age, and ethnicity.

Similarly, the mean differences in BMI‐for‐age percentile relative to the 95th percentile for intervention community relative to comparison community at 6‐ and 18‐month follow‐up were not significant (Table 3). Sensitivity analysis that excluded fifth graders (*n* = 201) or outliers (*n* = 16 children) did not change the estimates. Analysis evaluating effect modification by baseline BMI (with obesity vs. without), sex, and grade indicated no statistically significant differences between groups (data not shown).

Table 4 shows the change in BMI *z*‐score by exposure composite score in the intervention group at 6 months and 18 months. At 6 months, children who participated in more intervention components had greater reduction in BMI *z*‐score (0 = 0.07, > 0 to 0.25 = 0.009, > 0.25 to 0.5 = 0.002, > 0.5 to 0.75 = −0.04, and > 0.75 to 1 = −0.05). This relationship was statistically significant in models both unadjusted (*p*‐trend = 0.008) and adjusted for baseline BMI (*p*‐trend = 0.009). Similar trends were observed for the BMI‐for‐age percentile relative to the 95th percentile.

**TABLE 4:** Exposure composite score on primary outcome among intervention group (*N* = 279).

	0	1 (> 0–0.25)	2 (> 0.25–0.5)	3 (> 0.5–0.75)	4 (> 0.75–1)	*p*‐trend (unadjusted)	*p*‐trend, adjusted for baseline BMI
Composite score at 6 months, mean (SD)							
*N* (%)	38 (13.6)	26 (9.3)	106 (38.0)	58 (20.8)	51 (18.3)		
Baseline BMI	19.6 (3.9)	20.8 (5.8)	21.4 (5.4)	22.0 (4.6)	20.2 (4.1)	0.36	
BMI *z*‐score T2 change	0.07 (0.23)	0.009 (0.25)	0.002 (0.18)	−0.04 (0.22)	−0.05 (0.21)	0.008	0.009
BMI relative to 95th percentile T2 change	0.88 (3.48)	0.63 (4.35)	−0.30 (3.70)	−0.75 (4.28)	−0.95 (3.58)	0.012	0.011
Composite score at 18 months, mean (SD)							
*N* (%)	98 (35.1)	79 (28.3)	72 (25.8)	24 (8.6)	6 (2.2)		
Baseline BMI	22.0 (5.7)	21.0 (4.6)	20.2 (4.1)	19.0 (3.5)	21.8 (4.4)	0.006	
BMI *z*‐score T3 change	0.03 (0.32)	0.003 (0.32)	0.04 (0.32)	−0.07 (0.29)	0.04 (0.40)	0.55	0.41
BMI relative to 95th percentile T3 change	1.27 (6.69)	−0.48 (7.42)	0.17 (5.74)	−2.45 (4.51)	3.07 (8.27)	0.25	0.38

*Note:* Exposure composite score represents overall intervention exposure, ranging from 0 (no components received) to 1 (all four components fully received).

Abbreviations: BMI, body mass index; T, time.

## 4 Discussion

This trial examined the effectiveness of a multi‐level intervention on BMI *z*‐score in two rural, largely Hispanic communities. Between‐group comparisons showed no significant differences in BMI *z*‐score or BMI‐for‐age percentile relative to the 95th percentile at six‐ or 18‐month follow‐up. However, among intervention participants at six ^‐^month follow‐up, higher intervention exposure was significantly associated with reduced BMI *z*‐score and BMI‐for age percentile relative to the 95th percentile. This relationship was not maintained at 18 month.

Studies reporting the efficacy of community‐wide interventions in pediatric obesity prevention in the U.S. and abroad are small but growing [38, 39, 40]. Our study highlights both the promise and the challenges of implementing multi‐level interventions to address obesity in rural communities. The CBPR approach of engaging community partners through local priorities helped leverage resources, enhance community capacity, and build commitment. The community momentum created with STRIDE facilitated the post‐intervention adoption of Ciclovía as annual city programing and the creation of a planning guide that communities can use to implement their own Ciclovías [41]. The intervention community continues to leverage partnerships built during the intervention phase with farmworkers, parks and recreation, community‐organizations, and retailers to create an annual planning committee and use the planning guide as a roadmap to implement the Ciclovía [41]. This new infrastructure could serve as a platform to augment community programming to enhance PA, such as a rural community marathon.

Despite these successes, rural communities in the U.S. and internationally face unique structural and environmental barriers such as geographic isolation, limited transportation infrastructure, and long distances between homes, schools, and community resources. These factors influenced implementation and participation, particularly in the family‐level components that required scheduled attendance, even with the strong community relationships fostered through STRIDE. In contrast, the school‐based components achieved greater implementation success, building on an evidence base suggesting that interventions that leverage existing infrastructure such as schools may be more equitable and successful than those requiring additional family resources [21, 42]. This study also highlights the strengths of rural communities, including social cohesion and the role of schools in shaping community norms and fostering community ownership.

This study also detected a significant relationship between exposure to intervention components and BMI *z*‐score, where increased participation in the multi‐level components was significantly associated with reduced BMI *z*‐scores. It is plausible that intervention components worked synergistically, with each component reinforcing the others. For instance, the stories from the comic books may have reinforced the teachers' lessons. Similarly, the classroom PA breaks may have enhanced the PA messages children heard during Ciclovía. The synergistic nature of multi‐level interventions directed at different sectors of society, such as schools and news outlets, has been previously described [26, 43, 44, 45]. Despite the significant relationship between exposure and outcome, there was no overall significant intervention effect. Future research is needed to understand aspects of the intervention that were acceptable to children and their families, and components that might have been a burden for teachers and CHWs to deliver, impacting intervention fidelity.

STRIDE was designed as a community‐based obesity prevention intervention for Hispanic families in the rural U.S. by offering Spanish‐language materials, including culturally responsive images and resources, and engaging CHWs and teachers from the community. However, the approach has broader applicability for diverse rural populations internationally. For example, the CBPR approach, multi‐level intervention, and emphasis on school‐based intervention delivery may be applicable to other communities with similar structural characteristics. Such interventions would require adaptations specific to their communities, including dietary recommendations, PA preferences, and communication approaches.

This study has several important strengths. First, it is one of the first to focus on Hispanic children in a rural setting, where obesity prevention research remains limited. Second, the intervention leveraged authentic community engagement, with broad participation of community partners representing school leadership, community‐organizations, city councils, parks and recreation, and the police department to design and implement the intervention.

There were also several limitations to this study. The quasi‐experimental design may have introduced unmeasured confounders, potentially biasing the effect of the intervention. Additionally, the study was designed to evaluate the multi‐level intervention as a whole rather than to evaluate single component‐level effects. Thus, it is not possible to determine whether specific intervention components or behavioral foci contributed differentially to outcomes. The study team had limited control over the implementation of school‐level components. Although the teachers were trained and provided with materials for implementing the media literacy education and PA breaks including detailed instructions for delivering and tracking each component (e.g., number of PA breaks, poster tallies), delivery and documentation differed by teachers. Finally, family‐level components (Family Night attendance, family class participation) demonstrated lower engagement compared with school‐level components. These limitations reveal important lessons about intervention fidelity across levels.

Differences in engagement likely reflect structural barriers disproportionately affecting rural populations, such as limited public transportation, geographic isolation and long travel times, inflexible work schedules, and competing family and caregiving responsibilities. Furthermore, low participation in some of the intervention components suggests a need for a thoughtful redesign to make the intervention more accessible for this population. Future multi‐level intervention studies targeting obesity in rural areas may consider assessing implementation outcomes beyond effectiveness, such as feasibility, acceptability, and appropriateness to inform necessary adaptations at both component and whole intervention levels [46]. Future studies may also assess community readiness to deliver and engage in intervention components, which can help ensure that the intervention components are feasible within a given context [47].

## 5 Conclusion

A community‐based, multi‐level intervention did not show an overall significant effect on BMI *z*‐scores among rural Hispanic children. While there was a significant correlation between levels of engagement with intervention components and BMI *z*‐scores, the interpretation is limited due to the limitations of the study. The STRIDE study had similar intervention components shown to be successful in other community‐based trials, but it had limited programming on structural and policy changes as well as moderate engagement from parents and teachers. This study adds to the small but growing literature on community‐based multi‐level interventions involving CBPR. Given the enormous public health challenges of pediatric obesity, comprehensive strategies that involve changes in the environment can be a step toward turning the tide on this problem and a necessary direction for the future.

## Author Contributions

L.K.K. conceptualized the study, secured funding, provided study design and oversight, data collection, data analysis, manuscript preparation and revision. E.R.S. contributed to study implementation, data analysis, manuscript preparation, revision, and approval, and M.K. contributed to study design, data analysis, manuscript revision and approval. E.J. contributed to study design, data analysis, and manuscript revision and approval. S.H.J. contributed to data analysis, manuscript preparation, revision, and approval. J.A.M. contributed to data analysis and manuscript revision and approval. S.B. contributed to study implementation, data collection, and manuscript revision and approval. L.X. contributed to data analysis, manuscript preparation, revision, and approval.

## Funding

This work was supported by the financial support of the National Institutes of Health (Grant U01 MD010540). L.K.K. was partially supported by the National Center for Advancing Translational Sciences of the National Institutes of Health (Grant UL1 TR002319), and from the National Cancer Institute (Grant P30CA014089). The content is solely the responsibility of the authors and does not necessarily represent the official views of the National Institutes of Health.

## Conflicts of Interest

The authors declare no conflicts of interest.
